# Potential microglia‐based interventions for stroke

**DOI:** 10.1111/cns.13291

**Published:** 2020-02-16

**Authors:** Ling‐Zhi Li, Yu‐You Huang, Zhen‐Hong Yang, Si‐Jia Zhang, Zi‐Ping Han, Yu‐Min Luo

**Affiliations:** ^1^ Institute of Cerebrovascular Disease Research and Department of Neurology Xuanwu Hospital of Capital Medical University Beijing China; ^2^ Beijing Geriatric Medical Research Center and Beijing Key Laboratory of Translational Medicine for Cerebrovascular Diseases Beijing China; ^3^ Beijing Institute for Brain Disorders Beijing China

**Keywords:** aging, crosstalk, microglia, sex, stroke, therapy

## Abstract

A large number of families worldwide suffer from the physical and mental burden posed by stroke. An increasing number of studies aimed at the prevention and treatment of stroke have been conducted. Specifically, manipulating the immune response to stroke is under intense investigation. Microglia are the principal immune cells in the brain and are the first line of defense against the pathophysiology induced by stroke. Increasing evidence has suggested that microglia play diverse roles that depend on dynamic interactions with neurons, astrocytes, and other neighboring cells both in the normal brain and under pathological conditions, including stroke. Moreover, there are dynamic alterations in microglial functions with respect to aging and sex differences in the human brain, which offer a deep understanding of the conditions of stroke patients of different ages and sex. Hence, we review the dynamic microglial reactions caused by aging, sex, and crosstalk with neighboring cells both in normal conditions and after stroke and relevant potential interventions.

## 1 INTRODUCTION

Microglia are the predominant brain‐resident cells and mainly originate from the yolk sac.^1^, ^2^, ^3^ Distinct from macrophages, the population of microglia in the mature central nervous system (CNS) is maintained by self‐differentiation and self‐renewal.^4^, ^5^ They are in charge of maintaining brain homeostasis through immune surveillance and immediately executing active immune responses to pathological states in the CNS.^6^, ^7^ Stroke is a devastating disease with high morbidity and mortality worldwide. Recently, growing evidence has emphasized the role of the immune response in stroke, but how immune cells and their mediators are implicated in stroke‐induced neuroinflammation is still controversial.^8^, ^9^ Given that microglial cells are able to exert various functions under physiological or pathological conditions, diverse populations of microglia with different functional characteristics might be targeted for disease prevention or treatment after stroke. Here, we focus on microglial heterogeneity based on aging and sex differences and how microglia crosstalk with neighboring cells after stroke and discuss relevant potential microglia‐based interventions for stroke therapy.

## 2 AGING MICROGLIA AND RELEVANT INTERVENTIONS FOR STROKE

Aging is associated with increased CNS inflammation, which is largely manifested by dysfunctional microglia. Mounting evidence in *Macaca nemestrina* and rodents suggests that microglial activation is an age‐dependent process in the normal brain.^10^, ^11^, ^12^ There is an overall trend of microglial hyperreactivity in aged mice.^13^, ^14^ In humans, dystrophy and degeneration of microglia, which result in deterioration of the defensive and neuroprotective capabilities of the senescent CNS, are implicated in brain aging.^15^ In turn, aging is related to low‐grade neuroinflammation, including increased levels of released proinflammatory cytokines and upregulated expression of inflammatory markers on microglia.^16^ In addition, microglia in the aging brain undergo metamorphoses due to dystrophy, and the morphological features of the dystrophic cell population include deramification, fragmentation, and spheroid formation.^15^ Importantly, in aging‐related diseases, microglial activation varies nonlinearly with developmental age; thus, studies examining the role of microglia in senile diseases are of great significance.^17^

Stroke is a disease that mainly affects the elderly. In mice, aging has been proven to have a significant influence on stroke outcomes, which is likely associated with age‐specific inflammatory responses.^18^ Studies have demonstrated that aging is an important determinant of stroke outcomes and is positively correlated with worsened functional recovery after stroke.^19^ Different cellular and molecular responses to stroke in aged microglia versus young microglia have been reported. For instance, there are higher numbers of interferon regulatory factor 4 (IRF4)‐ and CD206‐positive microglia in the ischemic brains of young mice, whereas aged mice express more IRF5 and major histocompatibility complex class II (MHCII) on microglia. In addition, serum anti‐inflammatory cytokines, including transforming growth factor beta (TGF‐β), interleukin (IL)‐4, and IL‐10, are more prominently upregulated in young mice after middle cerebral artery occlusion (MCAO), whereas proinflammatory cytokines (TNF‐α, iNOS, and IL‐6) are more prominently upregulated in aged mice.^19^ Therefore, targeting aging microglia or rejuvenating them in stroke patients may represent a promising therapeutic strategy. Physical exercise after stroke was found to be a therapeutic approach targeting microglia in aged MCAO mice. Compared to mice in the control group, aged MCAO mice show a greater proportion of CD86‐ (a positive costimulatory molecule and an M1 marker) positive microglia. However, when allowed access to a running wheel, aged female and male mice show a decreased proportion of CD86‐positive microglia in the brain.^20^ It is reasonable that stroke patients may benefit from physical exercise, which may modify aged microglia. Lifestyle factors also account for age‐related neuroinflammation and microglial dysfunction.^21^ Studies have suggested that moderate exposure to environmental enrichment substantially reduces the damage caused by inflammatory microglia and is beneficial for patients with subcortical ischemic stroke and the resulting vascular dementia.^22^

The rejuvenation of aged microglia may help preserve neurological integrity and promote regeneration in the aging CNS after stroke. As aged microglia produce more proinflammatory cytokines than young ones during stroke, drugs that penetrate into the CNS to inhibit microglia, such as minocycline or laquinimod, might be beneficial in tempering the excessive proinflammatory response.^23^, ^24^ These drugs may partially impede the beneficial effects of microglia, such as positive phagocytosis,^25^ but therapy that preferentially enhances phagocytosis and chemotaxis is also effective. For instance, monophosphoryl lipid A modified from lipopolysaccharide is an agent that mainly stimulates TIR‐domain‐containing adapter‐inducing interferon‐β, but not the proinflammatory myeloid differentiation factor 88 pathway downstream of Toll‐like receptor (TLR4),^26^ and its effect has been proven to increase the microglial phagocytosis of amyloid‐β in Alzheimer's disease mouse model and improve clinical outcomes.^27^ Hence, a more desired therapy that alleviates the excessive release of proinflammatory cytokines from aging microglia while enhancing phagocytosis and chemotaxis may be more reasonable for stroke.

## 3 DIFFERENT SEXUAL MICROGLIA AND INTERVENTIONS FOR STROKE

Mounting evidence suggests that microglial function is different between males and females.^28^, ^29^, ^30^ Clear differences in microglial CNS colonization are exhibited during neurodevelopment. The sex differences of microglial morphology, density, and function among neonatal, juvenile, and adult mice support this hypothesis.^31^, ^32^, ^33^, ^34^ Specifically, several studies have reported that males have more microglia in select brain areas early in postnatal development, while females have more microglia with an activated/ameboid morphology later in development and in adulthood.^35^, ^36^, ^37^ Primary microglia from male rats migrate faster than those from females and show phagocytic capacity in vitro. Furthermore, microglial density varies among different regions varies based on sex in mice at different stages throughout life.^38^, ^39^

It is widely accepted that males and females respond to stroke differently. Biological sex influences many variables that are important in stroke, such as general health condition, cerebrovascular status, symptomatology, and therapeutic response.^40^ Moreover, a large body of evidence has indicated that the stroke‐induced activation of inflammation is sexually dimorphic. After cerebral ischemia, microglia in female mice exhibit a more robust anti‐inflammatory response than those in males, and male mice exhibit an increase in CD11b immunoreactivity after MCAO.^41^, ^42^, ^43^ Clinically, patients with ischemic stroke present sex‐related differences in 3‐month outcomes and mortality, and sex‐related differences in prehospital data, molecular markers, and clinical variables have been documented.^44^ Further research has demonstrated a marked deregulation of microglial activation in ovariectomized and/or ERα knockout mice and increased vulnerability to ischemic injury, indicating that microglial heterogeneity poststroke is sex hormone‐dependent.^45^ This has been validated in older, postmenopausal women. Furthermore, elderly women suffer from higher morbidity and mortality and exhibit poorer recovery after stroke than age‐matched men. In aged animals, studies have shown that sex chromosome complement may contribute to ischemic sensitivity and lead to sex differences in CNS immune responses.^46^ Correspondently, gene expression in microglia has been determined to be sexually divergent, and these differentially expressed genes in microglia are enriched in the complement components pathway.^47^ Furthermore, the sexually divergent C1q protein in microglia has been confirmed to account for the sexual dimorphisms of stroke.^47^

Sex has been gradually recognized as an important biological variable in the treatment of stroke, and sexually dimorphic microglia are potential targets. Currently, hormone replacement therapies are used to inhibit the activation of microglia to reduce neuroinflammation in mice.^48^, ^49^ Interestingly, the preferential inhibition of poly (ADP‐ribose) polymerase (PARP), such as with minocycline, can protect males but not females against neonatal ischemia by modulating microglial phenotypes.^50^, ^51^, ^52^

## 4 MICROGLIA‐NEIGHBORING CELLS CROSSTALK AND INTERVENTIONS FOR STROKE

In response to acute stroke, pandirectional microglia rapidly assemble in a unidirectional manner and move toward the injury site.^53^ The migratory ability of microglia is indispensable for brain homeostasis.^54^ However, the cellular and molecular mechanisms underlying this microglial migration, especially during pathological states, are not fully understood. In general, cell migration can be described as a series of events that require well‐coordinated molecular signals, based on which microglia interact with their neighboring cells. The crosstalk between microglia and other cells includes direct and indirect interactions. Direct interactions are defined as activities such as aggregation, adherence, and phagocytosis; indirect interactions are implemented by intercellular fractalkine signaling, secreted soluble factors, and extracellular vesicles.

### 4.1 Neuron‐microglia crosstalk in stroke

Microglia participate in inflammatory reactions throughout all phases of the stroke cascade, from acute neuronal cell death to later subacute stages. After stroke, the brain loses 2 million neurons and 14.8 billion synapses every minute.^55^ Due to the disruption of the neuronal cytomembrane, intracellular proteins/enzymes or nuclear DNA/RNA and cellular debris are released. Thereafter, damage‐associated molecular pattern molecules (DAMPs), such as ATP, S100, reactive oxygen species (ROS), and high mobility group box 1 (HMGB1), are released.^56^, ^57^, ^58^ Pattern recognition receptors expressed on resident microglia and infiltrating immune cells bind to these DAMPs, which initiate aseptic immune responses (produce inflammatory cytokines) in compromised tissue to further influence stroke pathology. Inhibiting this polarized microglial extension toward the stroke core prevents the expansion of the injury. Among DAMPs, S100B was identified as an M1 marker gene induced in a mouse model of MCAO, and it was found to exacerbate cerebral ischemia in a murine model of MCAO model and promote microglial migration and M1 polarization.^59^ Dead/dying cells may also release a repertoire of signaling molecules such as purines, which are decrypted as “find me” and “eat me” signals by surrounding cells, including microglia,^60^ triggering subsequent microglia‐mediated phagocytosis and neuroprotection.^61^ Additionally, stroke‐exposed/stressed neurons can release “help‐me” signals^62^ comprised of a group of molecular determinants, including chemokines and cytokines such as CX3CL1, lipocalin‐2 (LCN2), fibroblast growth factor 2 (FGF2), IL‐34, and IgG.^63^ In a subarachnoid hemorrhage (SAH) model, FGF‐2 was determined to be a promising therapy to reduce neuronal apoptosis, acting as a “help‐me” signal through the activation of the FGFR3/PI3k/Akt signaling pathway.^64^

Moreover, other extracellular signals derived from microglia are linked. CCL2, TNFα,^65^ and vascular endothelial growth factors are representative examples of these extracellular signals, which result in neurotoxicity, neuroprotection, blood‐brain barrier (BBB) leakage or angiogenesis.^66^ It is reasonable to speculate that there is a certain balance between DAMPs and these salutary signals, which may be regulated mainly by microglia, after stroke.^67^, ^68^ Translational research focused on microglia‐neuron crosstalk to bind related receptors or ligands for stroke therapy is warranted.

Microglia also interact with elements of synapses in an activity‐dependent manner. In vivo imaging studies have demonstrated the dynamic interaction of microglia with synapses in the mouse cortex (microglia frequently but transiently contact synaptic spines and terminals),^69^ where microglia rapidly respond to cues of neural activity and neurotransmitter release.^70^, ^71^, ^72^ These interactions are significantly prolonged following ischemic stroke and are associated with subsequent synaptic elimination.^69^ Microglia actively phagocytose synapses that need to be eliminated.^73^, ^74^, ^75^ The recognition process of synaptic elimination may likely occur through “find me” and “clear me” signals as well. Microglia digest synapses through invagination of their membrane followed by sinking of axonal pinches in their cytoplasm. Finally, microglia seal their membranes to transport the structures for subsequent degradation.^76^ Likewise, the process of trogocytosis also requires a well‐directed microglial process that moves toward compromised synapses.^77^ Dysfunction in this critical process may be linked to the sequelae of stroke and influence long‐term neurological recovery.^78^ The interaction between microglia and synapses in the context of stroke remains to be determined.

A better understanding of neuron‐microglia crosstalk signaling may help lead to novel therapeutic strategies for neuroprotection and neurorecovery. Emerging studies have shown that using mesenchymal stem cells to modulate the activation of microglia is a potent treatment for stroke.^79^, ^80^, ^81^ Additionally, studies of ischemic stroke have shown that extracellular vesicles (EVs) have immunomodulatory and neuroprotective properties. Thus, it is tempting to design a preclinical trial of EV injection into an ischemic stroke animal model to induce anti‐inflammatory effects through interactions with microglia.^82^ A study of miR‐124‐enriched EV administration is planned, and miR‐124 has been proven to shift microglia to the M2 phenotype to benefit neurogenesis.^83^ In addition, in virtue of this potent carrier, there will be more promising clinical studies of EVs transplantation to shape microglia after stroke in the near future. Ubiquitin‐specific protease 18 has been reported to reduce the levels of proinflammatory cytokines and JAK/STAT pathway members in oxygen/glucose‐deprived BV2 microglia and promote neurogenesis in MCAO mice.^84^ It has also inspired us to intervene with ubiquitin‐specific protease 18 to degrade inflammatory cytokines in microglia. Hypoxic preconditioning in an MCAO model remarkably promotes the transformation of M1 microglia to the M2 phenotype without significantly affecting peripheral immune cells.^85^ Intravenous immunoglobulins are another possible approach to hamper neuroinflammation in ischemic stroke. Their neuroprotective effects on ischemic stroke are mainly realized through switching microglia toward protective subtypes without having significant effects on infiltrating peripheral immune cells.^86^

### 4.2 Crosstalk between microglia and other neuroglia (astrocytes and oligodendrocytes) in stroke

Crosstalk between microglia and other neuroglia is still largely unclear. Several lines of experiments have described microglia‐astrocyte crosstalk in normal conditions. As an illustration of indirect interactions, astrocyte‐derived diffusible factors such as TGF‐β partly contribute to the switching of outward K^+^ currents in ameboid rat microglia during the period of maturation.^87^ Similarly, without physical contact with astrocytes, in vitro mouse primary microglial ramification can be induced by astrocyte‐derived soluble factors, including macrophage colony‐stimulating factor (M‐CSF), granulocyte‐macrophage colony‐stimulating factor (GM‐CSF), and purines. Moreover, astrocytes contribute to microglial morphology,^88^ and astrocytic regional density correlates with microglial regional density.^89^ These results suggest that the dynamics of the microglial process may be in part directed toward astrocytes, although robust evidence for this is lacking in the literature. In the CNS, the binding of microglial‐derived cytokines to calcium channel‐coupled receptors on astrocytes results in the activation of phospholipase enzymes that liberate arachidonic acid (AA) from membrane lipoproteins. Thus, the mobilization of AA has been suggested to be a useful biomarker of neuroinflammation.^90^ During hemorrhagic stroke, reactive astrocytes may modulate microglial ROS production and abate excessive inflammation.^91^ However, astrocytes have also been reported to accelerate the inflammatory response in coordination with activated microglia.^92^ In a model of transient middle cerebral artery occlusion (tMCAO), the expression level of IL‐33 rapidly increases in oligodendrocytes and astrocytes one hour after tMCAO. Flow cytometry analyses have also confirmed high expression levels of the receptor of IL‐33 (ST2) on microglia and that the expression level dramatically increases after stroke. ST2 deficiency in mice exacerbates brain infarction and neurological deficits, and intracerebroventricular infusions of IL‐33 attenuate brain infarction in the tMCAO model. These results strongly argue that IL‐33/ST2 signaling is a beneficial interaction between microglia and other neuroglia. In addition, in vitro studies on co‐culture systems have suggested that IL‐33/ST2 signaling can potentiate the expression of M2‐related genes, such as IL‐10, in primary microglia.^93^ The activation of signaling between microglia and other neuroglia leads to a protective microglia phenotype that enhances neuronal survival against oxygen and glucose deprivation. Furthermore, in vitro studies have revealed that IL‐33‐activated microglia can release IL‐10, which is critical for the neuroprotective effects of microglia (M2). Similarly, a study suggested that in vivo infusion of IL‐33 into IL‐10 knockout mice fails to provide neuroprotection against tMCAO. Therefore, activated glial cellular signaling enhances the expression of M2 polarization markers on microglia and impairs the expression of M1 polarization markers after tMCAO. A clinical study of 259 stroke patients demonstrated that higher interleukin‐33 levels are positively correlated with better prognosis.^94^ However, this conclusion was inconsistent with the findings of another clinical study on 175 patients with aneurysmal subarachnoid hemorrhage (aSAH); this study reported that increases in the level of serum IL‐33 predict a worse prognosis of aSAH.^95^ Regulating IL‐33/ST2 signaling to promote A2 astrocytes is promising. More importantly, whether IL‐33 can be used to predict long‐term outcomes in stroke patients warrants further clinical investigation and targeting glial cell IL‐33/ST2 signaling as treatment for stroke needs further intensive study.

Microglia‐released cytokines such as IL‐4 and TGF‐α have been recently reported to be salutary mediators for oligodendrocytes. IL‐4 receptors have been confirmed to be expressed on oligodendrocytes, and research has shown that the IL‐4/PPARγ signaling axis is responsible for this interaction, promoting oligodendrocyte differentiation and remyelination after brain injury.^96^, ^97^ TGFα and CXCL4 also protect oligodendrocytes against ischemic stroke,^98^, ^99^ and further studies have determined that this protective effect on oligodendrocytes is mediated by the downstream factor STAT3 and thus contributes to white matter integrity and remyelination and improves neurological recovery in the subacute stage after stroke.

### 4.3 Crosstalk between microglia and infiltrating peripheral immune cells (neutrophils and lymphocytes) in stroke

In stroke, DAMPs released by stroke‐affected tissue (neurons, microglia, and astrocytes) and the impaired BBB recruit peripheral immune cells, including neutrophils and lymphocytes, to roll, adhere, aggregate, and infiltrate.^100^ In addition, activated endothelial cells, microglia, and astrocytes express intercellular adhesion molecule 1 (ICAM‐1) and vascular cell adhesion molecule 1 (VCAM‐1) to help with leukocyte‐endothelial cell adhesion. Microglial activity can also facilitate the preservation of damaged blood vessels, helping to restore the integrity of the compromised BBB.^101^ Upon infiltration, invaders can be engulfed by local sentries. The process of phagocytosis has been observed in an animal stroke model in vivo*,* and activated microglial cells engulf infiltrating neutrophils at the periphery of infarction.^102^ The systemic blockade of the protein very‐late‐antigen 4 immediately and effectively inhibits the entry of neutrophils into the brain and interactions with microglia.^103^ Microglia phagocytose neutrophils as well, and the process is impaired in CSF1R^+/−^ microglia.^104^ Microglial depletion increases the number of neutrophils in ischemic brain tissue and augments brain injury.^105^ Neutrophils also have N0, N1, and N2 phenotypes, and the exact phenotype targeted by microglia is still unknown. Whether this direct interaction is time‐dependent warrants further investigation. Manipulating CNS‐periphery crosstalk to impair proinflammatory N1 neutrophils and amplify the effect of N2 neutrophils may be a feasible treatment approach for stroke.^106^

T and B lymphocytes play critical roles in cell‐mediated immunity and have been recognized as secondary roles during the response to stroke. However, little is known about the effects of lymphocytes in stroke. In peripheral immunity, T lymphocytes can respond to specific antigens by destroying infected cells themselves via CD8+ T cells or by releasing inflammatory cytokines via Tregs and CD4+ T cells. Interactions between PD‐1 and PD‐L1 on CD8+ T cells have been shown to cause T‐cell exhaustion or suppression. One study suggested that activated microglia express PD‐L1.^107^ In parallel, microglia/CD8+ T‐cell cocultures in which the PD‐1/PD‐L1 pathway is blocked results in increased production of IL‐2 and IFN‐γ. In animal research, forkhead box P3 (foxp3)+ Tregs in the cerebrum of rats inhibit the LPS‐induced M1 phenotype microglia, implying that Foxp3+ T cells may inhibit neuroinflammation, possibly through shifting microglial phenotypes.^108^

Another study further confirmed that Tregs can shift the microglial response toward the M2‐like phenotype through the IL‐10/GSK3β/PTEN axis in vitro and in vivo in an intracerebral hemorrhage model.^109^ Similarly, a study demonstrated that poststroke microglia suppress the anti‐inflammatory function of infiltrating Tregs. A novel mechanism of microglial immunoregulatory activity through the Sirtuin2/HIF‐1α pathway was identified.^110^ Recently, a study reported that the CD3+CD4‐CD8‐T‐cell (double‐negative T cell; DNT) population is significantly upregulated in the peripheral blood of acute stroke patients. It is intriguing that infiltrating DNTs are located close to the microglial population in the ischemic brain territories of both MCAO mice and stroke patients. Furthermore, DNT‐intrinsic PTPN2 and FasL may collaboratively regulate the level of M1 microglia via the production of TNF‐α, which amplifies neuroinflammation and exacerbates brain injury after stroke.^111^ Therefore, infiltrating lymphocytes are critical driving forces for modulating microglia‐mediated neuroinflammation and ischemic brain injury.^112^ These findings highlight the potential for targeting microglia‐lymphocyte interactions for the development of therapies to reduce CNS insult, including stroke.^113^

**Figure 1:**
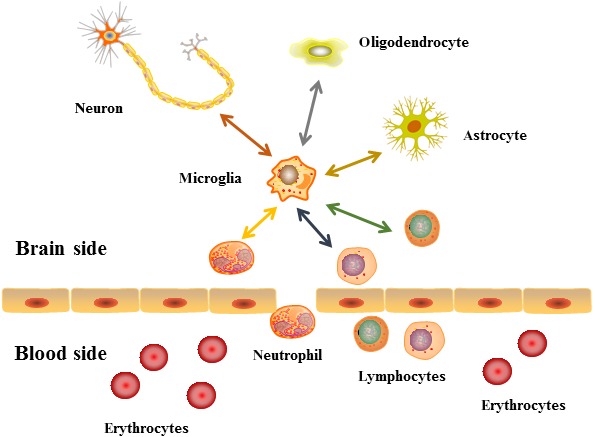
A diagram of crosstalk between microglia and neighboring cells. This sketch shows the existing crosstalk between microglia and neighboring cells (all cell types are labeled), and the arrows represent the interactions. The specific details of the crosstalk are listed in Table 1

Considering the findings that the severity of stroke is associated with the compromised areas, age and sex of patients, microglia cells are able to exert various functions under physiological and pathological conditions (Figure 1). A better understanding of microglial crosstalk with neighboring cells in compromised areas of the brain is required for the future development of targeted therapies for stroke (Table 1). Furthermore, a deeper investigation of microglia in aging and between the sexes may show great promise for future personalized medicine for stroke prevention and treatment.

**Table 1:**
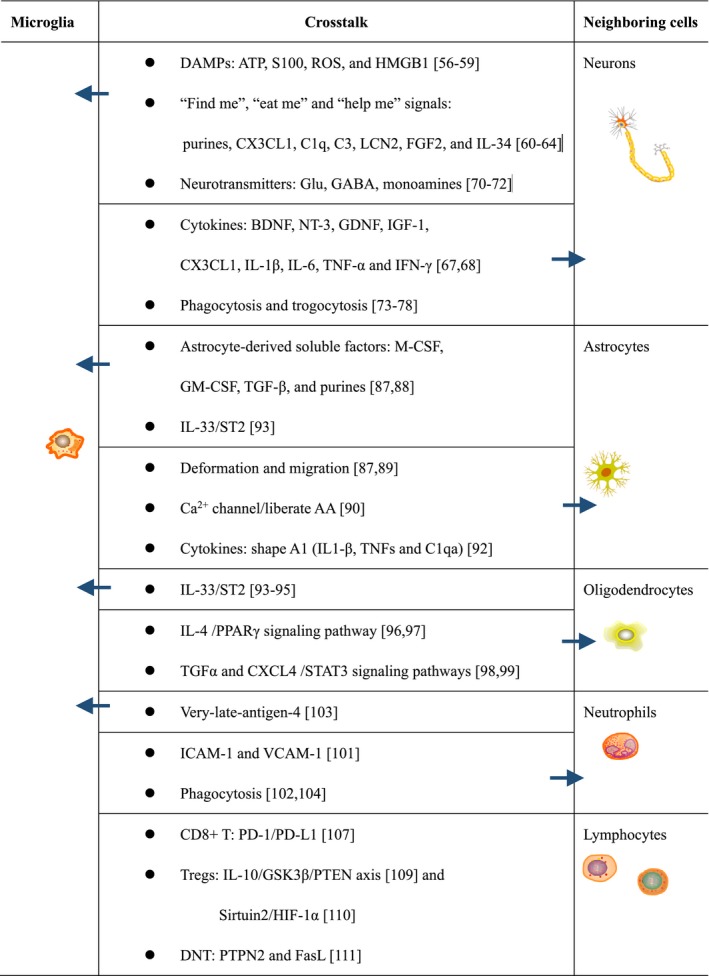
Crosstalk between microglia and their neighboring cells

Note

This table is a guide for Section 4. The numbers in brackets refer to the relevant references.

## CONFLICT OF INTEREST

The authors declare no conflict of interest.
